# Outcome of Incus Interposition after Preservation in Soft Tissue

**Published:** 2017-03

**Authors:** Mohammad Faramarzi, Sareh Roosta, Mahboobe Dianat

**Affiliations:** 1 *Otolaryngology Research Center, Shiraz University of Medical Sciences, Shiraz, Iran.*; 2 *Department of Otolaryngology, Shiraz University of Medical Sciences, Shiraz, Iran.*

**Keywords:** Autologous grafts, Chronic suppurative otitis media, Incus interposition, Ossiculoplasty

## Abstract

**Introduction:**

The lenticular process of the incus succumbs to necrosis in chronic otitis media. Few researchers have addressed the issue of autograft incus preservation in the soft tissue of the tragus or mastoid cavity. Nonetheless, preservation of the incus in this method during the second stage of ossiculoplasty is a subject that is still up for debate. This study was carried out to demonstrate the hearing outcome after a modification of the incus interposition technique, which involved preserving it in the periauricular soft tissue.

**Materials and Methods:**

In the primary operations, tympanoplasty was performed with a postauricular incision. At the end of the surgery, a small pocket was created to preserve the incus beneath the temporalis fascia. The second stage of ossiculoplasty was performed 6 to 18 months after the primary operation. Post-operative pure tone audiometry was analyzed after at least 12 months and was considered successful after achieving an air-bone gap (ABG) within 20 dB.

**Results:**

In this paper, we analyzed 199 ears. The mean duration of follow up was 2.5 years. We achieved post-operative ABG within 20 dB in 157 patients (78.9% of patients).

**Conclusion:**

This study indicates the efficacy and safety of incus interposition when it is preserved in the postauricular soft tissue.

## Introduction

Chronic otitis media is one of the most common diseases and the incus is the most necrotic ossicle that demands ossicular chain reconstruction (OCR) (1-4). The lenticular process of the incus is vulnerable to necrosis because it has a proximal narrow pedicle (5). In this situation, incus interposition is one of the most common options for OCR. This refers to using a sculptured incus to connect the stapes into the handle of malleus (6). When the stapes is present and the incus is normal in body size and bulk, autogenous incus is our first priority for OCR. However, the main drawback of using autogenous incus during second-stage incus interposition is the need for a bone bank. Moreover, disinfection techniques may result in the weakening of the ossicle (7).

Due to limitations of bone banks, an alternative solution is gaining popularity particularly in developing countries where expensive allografts are a real hindrance for OCR. This solution attempts to preserve the incus in the postauricular soft tissue for the second stage OCR. In 2007, Gyo et al. reported hearing results, in 17 ears, from preserving the incus within the mastoid cavity. In this study, only seven cases were followed up 5 years after OCR (8). Additionally, in 2010, Fritsch et al. described a preserving technique of autograft incus within the soft tissue of the tragus (9).

Due to the possibility of fixation and resorption, the durability of this method is debatable. We have attempted to show the effectiveness in preserving the incus in the postauricular soft tissue as an appropriate method for OCR.

## Materials and Methods

This retrospective study was performed in Dastgheib Hospital, a tertiary care hospital for otologic surgery in the south of Iran. This study was approved by the local institutional review board of Shiraz University of Medical Sciences.

From March 2003 to November 2014, all patients whose incus was preserved into the postauricular soft tissue for second stage autogenous incus interposition (AII) were included in this study. 

In order to reduce or even eliminate the effects of other prognostic variables, our criteria for inclusion were all patients who underwent canal wall down mastoidectomy due to extensive cholesteatoma in their primary operation, normal contralateral ear, and a follow-up of at least 12 months after second stage operation. Furthermore, other inclusion criteria were the presence of normal mucosa in the middle ear, presence of malleus, stapes, and incus with normal body size and bulk, in the second stage. An exclusion criterion was patients who did not complete their follow-ups.

All surgeries were performed by the chief author. The data, including age, sex, condition of the middle ear mucosa and situation of the ossicles, preoperative, and postoperative audiograms were recorded. 

In the primary operations, after complete removal of the cholesteatoma tissue, a 0.5-mm-thick silastic sheet was placed in the middle ear cavity. Then, temporalis fascia was used as an underlay tympanic membrane graft. At the end of the surgery, if the necrotic incus had a normal body size and bulk, it was selected for AII. In order to further assess the eroded incus for cholesteatoma involvement, the surgeon carried out a careful gross examination with an operating microscope. If it required surface stripping, such a procedure was performed. Then, a small pocket was created beneath the temporalis fascia, and it was inserted in the soft tissue pocket. Finally, the post auricular wound was closed in two layers.

The planned second stage AII was performed between 6 to 18 months after the primary operation. The ossicular chain was reconstructed with either the preserved incus or other materials, depending on the shape of the preserved autogenous incus and stapes. When the stapes capitulum was normal and the preserved incus had normal body size and bulk, it was remodeled and inserted between the stapes and the handle of the malleus. The technique detail of sculpting the incus has been illustrated elsewhere (10).

For evaluating hearing outcomes, pre-operative and post-operative pure tone audiometry in 0.5, 1, 2, and 4 kHz frequencies were analyzed. In our center, 3 kHz frequency is not evaluated. Therefore, in order to conform to the guidelines of the American Academy of Otolaryngology-Head and Neck Surgery Committee of Hearing and Equilibrium, we calculated the outcome of the hearing results as the mean of that of the 2 and 4 kHz frequencies. Also, pre-operative and post-operative word recognition score (WRS), and speech reception threshold (SRT) were evaluated. The surgery was considered successful when a postoperative air–bone gap (ABG) within 20 dB was achieved.

A paired t-test or a Willcoxon test was used in order to compare the preoperative and postoperative audiograms. Also, all analyses were performed using SPSS (version15; SPSS Inc., Chicago IL, USA). P-values less than 0.05 were considered significant.

## Results

At first, two hundred and seventy-five charts were reviewed, 32 (11.6%) of which were excluded because of incomplete or inadequate documentation and follow-up. 

During second stage surgery, the preserved autogenous incus was easily visible in the soft tissue pocket in all 243 cases. All of them were wrapped in a fine layer of mucosa. We did not detect any adverse reaction such as the granulation of tissue or cholesteatoma.

The stored incus stayed in an acceptable bulk in 216 cases (88.9%), apart from 27 cases (11.1%) in which it was subject to atrophy and was so feeble and flimsy that we could not sculpt it. 

On the other hand, in 17 cases (6.9%) superstructure of the stapes faded away between stages. Finally, the hearing results of 199 patients who met the inclusion criteria and underwent planned second stage AII, were analyzed.

In the study, there were 199 ears belonging to 199 patients including 59 (29.6%) male and 140 (70.4%) female. The patients’ age ranged from 15 to 67 years (mean: 34.3±12.3). Operation was performed on 92 left ears and 107 right ears. The minimum duration of follow up was 1 year and the maximum was 6.5 years (mean:2.5years). The hearing outcome, observed one year after ossiculoplasty, was used for analysis.We found that the mean pre-operative and post-operative AC was 44.3±9.3 dB, 31.0±9.7 dB, respectively (P<0.0001). The post-operative AC gain was 13.3dB. In addition, our results showed that the mean pre-operative BC was 10.8±5.0 and the mean postoperative BC was 9.4±5.7 (P=0.009). This result was statistically significant; however, it was not clinically significant. The post-operative BC gain was 1.4dB. As shown in Table 1, the mean pre-operative ABG was 24.4±8.4 dB and the mean post-operative ABG was 16.0±6.9 dB (P<0.0001). The post-operative gain in ABG was approximately 8.4 dB. When compared, pre and post-operative ABG in all frequencies were less than 0.0001.

**Table 1:** Preoperative and postoperative ABG and postoperative ABG gain at 0.5 to 3 kHz

	**0.5 kHz**	**1 kHz**	**2 kHz**	**3 kHz**	**Mean 0.5-3 kHz**
Pre-operative ABG	37.5±13.3*	36.6±13.7	35.6±11.3	30.6±11.8	24.4±8.4
Post-operative ABG	23.7±11.1	22.1±11.2	18.9±10.3	21.5±10.7	16.0±6.9
ABG gain	13.8±15.1	14.5±15.1	18.7±14.7	9.1±14.9	8.4±10.1

* Values are Mean ± SD, ABG: Air-Bone Gap

As shown in Table 2, approximately 157 patients (78.9%) obtained Post-operative ABG within 20 dB.

**Table 2:** Post-operative ABG distribution

**ABG distribution **		
dB	N	%
≤10	39	19.6%
11-20	118	59.3%
21-30	33	16.6%
>30	9	4.5%

ABG: Air-Bone Gap

In this study, the mean pre-operative and post-operative SRT was 44.7±11.1 dB and 30.1±11.3 dB(P<0.0001).The mean change of improvement in SRT was 14.6 dB. We found that the mean pre-operative and post-operative WRS was 96.9±5.9% and 98.1±7.0%, respectively. However, it was neither statistically nor clinically significant (P=0.065). The mean change in WRS was 1.5%. We found that 44 cases (22.1%) obtained a post-operative SRT result of more than 30 dB. Out of these 44 cases, 32 (72.7%) referred for revision OCR. Intra-operative findings in this group included: severely adhesive tympanic membrane in 14 cases (31.8%), reabsorption of incus in 11 (25%) patients, and 7 cases (15.9%) with bony fusion of incus to facial nerve canal in revision OCR.

## Discussion

  We presently have solid evidence that AII can provide satisfactory hearing results when preserved in the postauricular soft tissue pocket for second stage OCR. In this study, the ratio of closure of post-operative ABG≤ 20 in AII was 78.9%. In order to create an ideal condition for OCR (i.e. a dry normal middle ear mucosa and intact tympanic membrane) we propose planned second stage incus interposition. Other studies support this idea in the presence of cholesteatoma, adhesive otitis media, and cholesterol granuloma (6,8).

The ratio of post-operative closure of ABG≤ 20 has been reported as 57% to 79% in other series (11-15). Our result is in agreement with numerous reports and within the upper limit of the achieved range. Nevertheless, there have been a few studies specifically about the OCR with AII. In a similar review, conducted by O’Reilly et al., 137 patients who underwent OCR using autologous or homologous incus interposition were evaluated. They achieved 66.4% post-operative ABG≤ 20 dB and they did not find any extrusion, at the same time, the hearing results remained stable over time (11). In a study by Farrior and Nichols, they attained a hearing improvement with an ABG≤ 15 in 59% of patients (16). Nikolaou et al. accomplished a post-operative ABG of less than 20 dB in 74% of patients (17). This ratio has been recorded from 68% to 89% for titanium PORP (18-26). Some authors believe that there is no difference between titanium PORP and AII (15,27,28). On the other hand, Felek et al. found better results with AII and glass ionomer cement than plastipor or hydroxyapatite PORP (13). Somers et al. asserted that there was no difference between hydroxyapatite bone cement for repairing the incudostapedial joint and incus interposition regarding post-operative ABG≤ 20 (12). In a survey by Goldenberg and Emmet, they documented that 25% of North American surgeons selected autograft or homograft bone as their first choice in OCR. In addition, the satisfaction rate was 96% (29). In a UK survey, 86% of the surgeons applied allograft materials and 64% of them utilized autografts (30). Overall, this is a controversial issue among otolaryngologists.

We believe that the preservation of the incus in the postauricular soft tissue pocket is a safe technique. Previously, Fritsch and Moberly had found that the posterior tragus was an ideal site. They applied this technique in 16 cases and they confirmed that there were no failures to yield intact ossicles during the second stage (9). In another study, Gyo et al. used mastoid storage technique and found it as an effective option for planned stage OCR. All of their patients suffered from extensive middle ear cholesteatoma in the primary operations. They reported involvement of the incus with granulation tissue in 12 out of 24 ears (8). In addition, Wake et al. had a similar experience regarding residual cholesteatoma. Nevertheless, they did not find residual cholesteatoma in the mastoid cavity where the autogenous incus had been stored (31). On the other hand, in both studies bony adhesions of the autogenous incus had occurred and sometimes drilling was used for its removal (8,31). On the contrary, we did not find any cases that involved incus with granulation tissue or adhesions to the surrounding soft tissue. Therefore, the pathological condition of the mastoid cavity has no effect on the saved ossicles. One of the advantages of our approach is that there is no need for an additional incision because the same incision is required for the second stage OCR. Moreover, it would be easy to find it at the upper limit of incisions. Undeniably, we are aware of the drawbacks using AII. One is the relatively prolonged operation time. In addition, some authors noted a 1 to 83% rate of remodeling during the histological review of ossicular grafts and cortical bone grafts. However, they did not observe any reabsorption of the autologous bone graft (32,33). On the other hand, Nikolaou et al. noticed 12.5% of bone reabsorption in the incus interposition surgery (17). We also detected a reabsorption rate of 14.43%, which is similar to the above mentioned studies. Histopathological observations of ossicular grafts showed that autograft incus and malleus conserved their configuration and size for up to 25 years after OCR (34). Some authors found that there was no significant change in AII regarding post-operative ABG after 10 to 15 years (35). These data show that autologous bone grafts remain stable over time. The other theoretical disadvantage of AII is recurrence of cholesteatoma. Some authors believe that the operating microscope is not a reliable tool to decide if ossicles can be applied in OCR following chronic otitis media with cholesteatoma. They affirmed that persistent of squamous epithelium or cholesteatoma particles over ossicles that cannot be observed with the operating microscope may result in recurrence of cholesteatoma and failures of surgery (36,37). On the contrary, others asserted that when there is no macroscopic involvement of ossicles with epithelium, it does not influence OCR results (8,38). Our findings are consistent with this idea, because we did not observe any local recurrence of cholesteatoma at the site of preservation. Autogenous incus is also prone to resorption when the thermal injury is applied by drill during the sculpturing. We believe that one of the causes of incus necrosis may be this phenomenon. Therefore, as recommended, copious irrigation is mandatory to avoid such a problem (34). In addition, to eliminate thermal injury, we strongly recommend that surgeons use a sharp diamond burr during the sculpturing.

One of the strengths of this study was the medium-term results. These are certainly encouraging and demonstrate the reliability and effectiveness of this preservation method. A limitation in this study was the retrospective nature of this investigation which raises the possibility of selection bias.

The important clinical lesson of this study is generating a new concept that preserves the incus in the postauricular soft tissue pocket which may be a valuable alternative for OCR. Furthermore, our results address ways in which OCR can be made more cost-effective and approachable.

## Conclusion

In this study, we have evaluated hearing results in a large group of patients who underwent planed stage AII. The incus was preserved in the postauricular soft tissue pocket. A success rate of 78.9% in a follow-up of 1 to 6.5 years indicates the efficacy and safety of this method. We recommend further multicenter randomized studies to prove the efficacy of this method in ears presenting with extensive cholesteatoma.
